# 

**Published:** 1878-12

**Authors:** 


### CONTENTS.

ORIGINAL.

ART. I.—Pulmonary Consumption. By Willis E. Ford, M. D., Assistant Physician N. Y. State Lunatic Asylum, Utica, N. Y., .... 161

ART. II.—Iodide of Potassium in Syphilis. By Prof. J. F. Miner. Reported with private surgical cases, by C. A. Wall, B. S, a member of the class, .... 173

MISCELLANEOUS.

The treatment of Ulcers with Saturated Solution Chlorate Potassium. By T. M Rochester, M. D., Brooklyn, N. Y., .... 177

Dialysed Iron, .... 181

Mortality of Doctors, .... 183

Chloral Anæsthesia in Children, .... 183

The Illinois State Board of Health, attacking Quackery, .... 184

Surgeon-General of the Navy .... 185

List of Physicians who have died of Yellow Fever in Memphis, .... 186

Artificial Respiration, .... 187

Nitrate of Silver as a Uterine Caustic, .... 187

Dr. Grissom's charges against Dr. Hammond, .... 188

CORRESPONDENCE.

Letter to the Journal, .... 190

EDITORIAL.

Suits for Malpractice, so common as to constitute an Epidemic, .... 192

Maltine, .... 193

Items, .... 193

Hamilton on Nervous Diseases, .... 194

Patent Spring Washer Axles, .... 194

List of Exchange Journals, .... 195

BOOKS REVIEWED:

Cyclopedia of the Practice of Medicine, Vol. XIII .... 196

Playfair's System of Midwifery, .... 198

Books and Pamphlets Received, .... 200

